# Photosensitizers Based on G-Quadruplex Ligand for Cancer Photodynamic Therapy

**DOI:** 10.3390/genes11111340

**Published:** 2020-11-12

**Authors:** Keiko Kawauchi, Ryoto Urano, Natsuki Kinoshita, Shin Kuwamoto, Takeru Torii, Yoshiki Hashimoto, Shinya Taniguchi, Mitsuki Tsuruta, Daisuke Miyoshi

**Affiliations:** Frontiers of Innovative Research in Science and Technology, Konan University, 7-1-20 Minatojima-minamimachi, Chuo-ku, Kobe, Hyogo 650-0047, Japan; kawauchi@konan-u.ac.jp (K.K.); mha.lily.ryoto@gmail.com (R.U.); sayo2410rin.orange@gmail.com (N.K.); kuwa.jhy@gmail.com (S.K.); grignard.tor2@gmail.com (T.T.); 5678hasimoosan@gmail.com (Y.H.); shinya.taniguchi.ngi@gmail.com (S.T.); ikustimand923@gmail.com (M.T.)

**Keywords:** G-quadruplex, photodynamic therapy, photosensitizer, cancer, telomeres, *RAS*

## Abstract

G-quadruplex (G4) is the non-canonical secondary structure of DNA and RNA formed by guanine-rich sequences. G4-forming sequences are abundantly located in telomeric regions and in the promoter and untranslated regions (UTR) of cancer-related genes, such as *RAS* and *MYC*. Extensive research has suggested that G4 is a potential molecular target for cancer therapy. Here, we reviewed G4 ligands as photosensitizers for cancer photodynamic therapy (PDT), which is a minimally invasive therapeutic approach. The photosensitizers, such as porphyrins, were found to be highly toxic against cancer cells via the generation of reactive oxidative species (ROS) upon photo-irradiation. Several porphyrin derivatives and analogs, such as phthalocyanines, which can generate ROS upon photo-irradiation, have been reported to act as G4 ligands. Therefore, they have been implicated as promising photosensitizers that can selectively break down cancer-related DNA and RNA forming G4. In this review, we majorly focused on the potential application of G4 ligands as photosensitizers, which would provide a novel strategy for PDT, especially molecularly targeted PDT (mtPDT).

## 1. Photodynamic Therapy for Cancer

**Figure 1:**
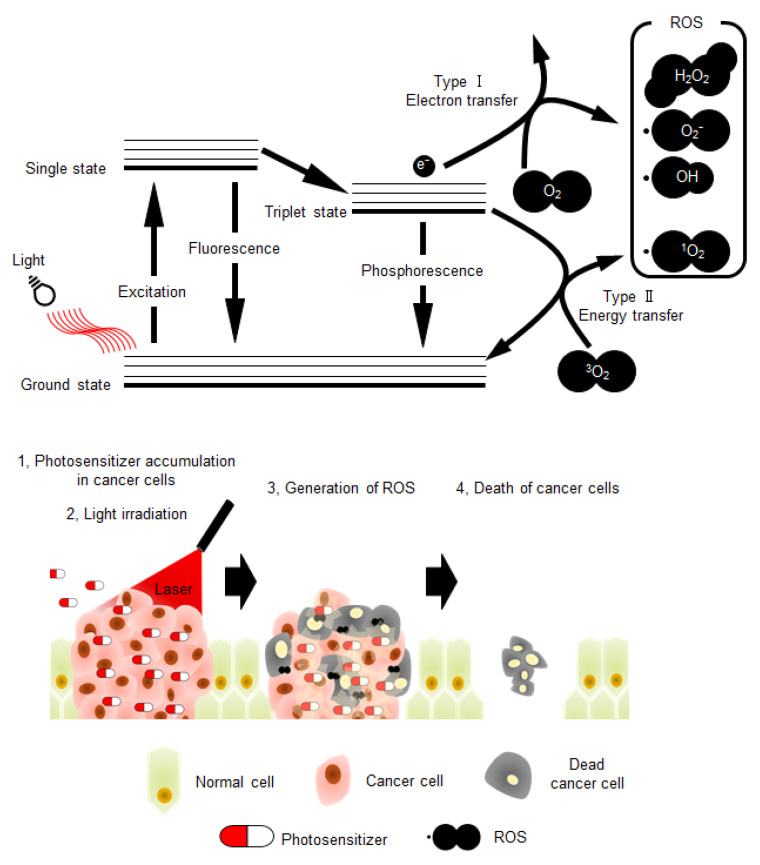
The schematic illustration of photochemical reactions in photodynamic therapy (PDT), and schematic model depicting the PDT strategy. ROS: reactive oxidative species.

Photodynamic therapy (PDT) is considered as a minimally invasive cancer treatment compared with other major cancer therapies, including surgery, chemotherapy, and radiotherapy [1,2]. In some cancer types, such as skin and lung cancers, tumor growth is especially confined to the surface of the tissue, and thus the effect of PDT is comparable with that of other major treatments [2,3,4]. Briefly, the procedure of PDT (Figure 1) involves administration of the photosensitizer in the body. Subsequently, photo-irradiation using near-infrared light with wavelength of 600–800 nm is directed toward the cancer tissue to activate the photosensitizer. The electronic energy of photo-activated photosensitizer is transferred to the surrounding substrates, such as biomolecules (type I reaction) or triplet state molecular oxygen (^3^O_2_) (type II reaction), resulting in reactive oxidative species (ROS) production [5]. Type I reaction generates radicals and radical anion species (e.g., O_2_^•−^, HO^•^); however, type II reaction produces singlet oxygen (^1^O_2_). These two competing mechanisms can occur simultaneously, leading to excess ROS production and cytotoxicity in cancer cells. Therefore, photosensitizers play a central role in PDT, and their characteristics highly influence the effects of this therapy. Moreover, to avoid side effects in normal tissues and enhance therapeutic effects, the photosensitizer should be activated by light corresponding to an absorbance band in the near-infrared region [4,6]. This is because endogenous chromophores are activated by light with wavelengths shorter than that of near-infrared light [2,7]. Additionally, the important properties of photosensitizers that need to be considered include low toxicity in the absence of photo-irradiation and high accumulation rate in cancer tissues (Figure 1) [8,9]. The main reason for the high accumulation rate of photosensitizers in cancer tissues is their high affinity towards low-density lipoprotein receptors (LDLRs), which are overexpressed in most of the cancer cells [10,11].

## 2. Properties of DNA and RNA G4s

**Figure 2:**
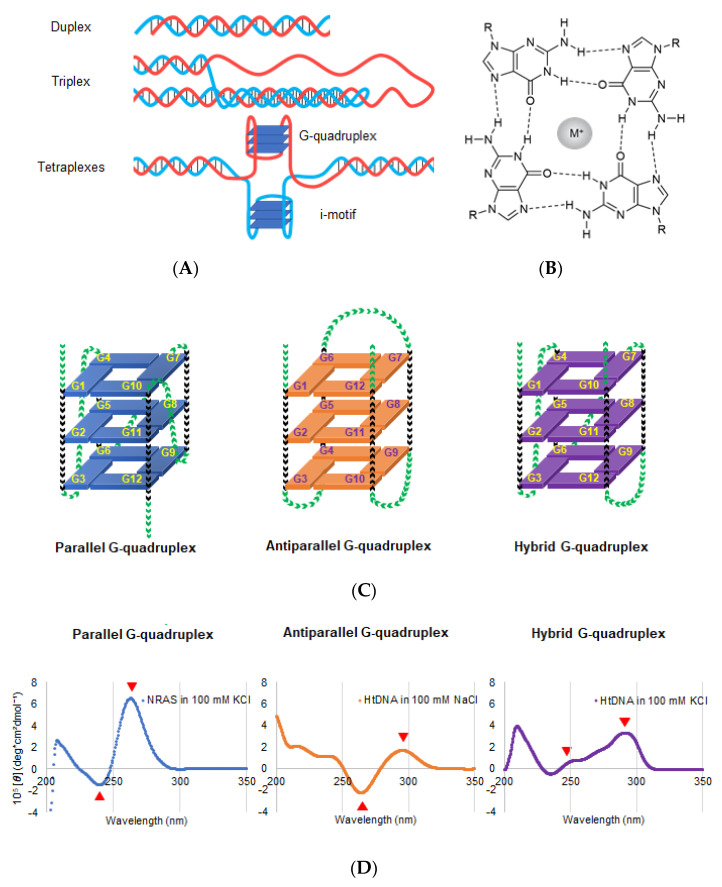
(**A**) Schematic DNA structures; duplex (upper), triplex (middle), and tetraplexes (lower). Tetraplexes including G-quadruplex and I-motif. (**B**) Chemical structure of G-quartets along with the cation coordinates of O6 sites corresponding to four guanines. The sphere with M^+^ in the center of G-quartet represents a central cation. (**C**) Schematic structure depicting the G-quadruplex (G4) folding conformation: parallel (left), antiparallel (middle), and hybrid (right). Green and black arrowheads indicate the loop and guanine region, respectively. (**D**) Typical CD spectra of the parallel, antiparallel, and hybrid conformations. Left: CD spectrum of NRAS DNA, d(GGGAGGGGCGGGTCTGGG), forming parallel G4 in a buffer containing 100 mM K^+^. Middle: CD spectrum of telomeric DNA, dA(GGGTTA)_3_GGG, forming antiparallel G4 in a buffer containing 100 mM Na^+^. Right: CD spectrum of telomeric DNA of, dA(GGGTTA)_3_GGG, forming hybrid G4 in a buffer containing 100 mM Na^+^ (right). Red arrows indicate the representative peaks of each G4 conformation.

Although nucleic acids form canonical duplex structures, they can potentially fold into non-canonical structures depending on their nucleotide sequence and molecular environment [12]. G-quadruplex (G4) is the representative non-canonical structure of DNA and RNA, which usually comprises guanine-rich sequences (Figure 2A) [13,14]. The most well-known guanine-rich sequence is observed at telomere region at the chromosome ends. In humans, the telomeric region is composed of repetitive guanine-rich sequence, (TTAGGG)_n_, and its complementary cytosine-rich sequence, (CCCTAA)_n_. Four guanine bases of the guanine-rich sequences are associated with Hoogsteen-type base pairs to form a large coplanar known as G-quartet (Figure 2B) [13,14]. This large coplanar surface with four guanine bases is distinct from other nucleic acid structures and thus forms a suitable structural platform for ligand binding. Stacking interactions between two, three, or four G-quartets and coordination interactions between guanine O6 atoms and cations likely contribute to the formation of thermodynamically stable G4 structures (Figure 2C) [15].

Besides the telomeric region, G4-forming sequences exist abundantly in the promoter and UTR of cancer-related genes [16]. The consensus G4-forming motif, G_3–5_N_1–7_G_3–5_N_1–7_G_3–5_N_1–7_G_3–5_, where N refers to any nucleotide, exists at approximately 376,000 sites in the human genome [17]. In addition to the G4 derived from the consensus sequence, it has been demonstrated that numerous G4-forming sequences, which form with two G-quartets and/or long loop regions, exist in the human genome [18], although these sequences do not always form G4. For example, various chemical environmental factors such as cation, pH, and molecular crowding conditions have been shown to affect the thermodynamic stability and strand orientation of G4 [14,19,20]. Taking into consideration the polymorphic nature of G4, it is well recognized that G4s are stabilized by cations in the following order: K^+^ > Na^+^ >> Li^+^ [19]. Generally, the intracellular concentrations of K^+^ and Na^+^ are 140 mM and 12 mM, respectively [2]; however, they largely depend on the cellular status. During tumor progression, K^+^ intracellular concentration decreases whereas Na^+^ intracellular concentration increases owing to the expression and activity of their specific ion channels/transporters and pumps [21]. Given that the G4 stabilizing ability of K^+^ is stronger than that of Na^+^ and that Na^+^ intracellular concentration is consistently lower than that of K^+^ even in cancer cells, changes in intracellular concentration of K^+^, rather than Na^+^, might affect G4 formation during tumor progression. Indeed, we have shown the possibility that a decrease in K^+^ intracellular concentration causes attenuation of G4 foci formation in the nucleus during tumor progression [22].

The polymorphic nature of G4 makes it distinct from other nucleic acid structures. Additionally, G4 can fold into different types of conformations, which includes parallel, antiparallel, and hybrid orientations of the four strands (Figure 2C) [23]. In the parallel conformation, all four strands are oriented in the same direction (Figure 2C, left panel). Its consensus sequence corresponds to the total number of nucleotides within the first and second loops being smaller than four [24]. In the antiparallel conformation, two strands are oriented in the opposite direction from the remaining two strands (Figure 2C, middle panel). It is known that only a few DNA sequences, such as *Oxytricha nova* telomere d(G_4_T_4_G_4_)_2_ and thrombin binding aptamer d(G_2_T2G_2_TGTG_2_TTG_2_), fold into the antiparallel conformation [25]. In the hybrid conformation, three strands go in one direction and one in the other (Figure 2C, right panel) [26,27]. The hybrid conformation was reported for the human telomeric sequence (TTAGGG)_2_ [27]. Unlike DNA G4s, almost all RNA G4s are monomorphic and adapt to the parallel conformation [24,28]. Moreover, an RNA G4 is thermodynamically more stable than its DNA counterpart. Therefore, these monomorphic and stable RNA G4s are promising targets for drug development.

Structural information, such as strand orientation, obtained from nuclear magnetic resonance spectroscopy (NMR) and X-ray crystallography, is important for designing ligands and drugs targeting G4 [29,30,31]. Additionally, circular dichroism (CD) spectrum is useful and convenient to identify different G4 conformations (Figure 2D). The parallel conformation has negative and positive peaks at 240 and 260 nm, respectively, whereas the antiparallel conformation has negative and positive peaks at 260 and 290 nm, respectively (Figure 2D). The hybrid structure has positive peaks at 245 nm and 290 nm [29,30,31,32].

## 3. Potential DNA or RNA G4s in Molecularly Targeted PDT

### 3.1. Telomeres

Telomeres are specific nucleoprotein complexes that are formed at the terminals of chromosomes, contributing to genome integrity [33]. The average length of a telomere in humans is approximately 10,000 bp at birth, which shortens naturally during DNA replication as 50–200 bp of DNA sequence at the 3′-end is not replicated in each progressive cycle [34]. Telomere shortening is known to be accelerated by stresses such as oxidative stress and DNA damage agents; however, the underlying molecular mechanism is not completely understood [35]. Moreover, it leads to cellular senescence, which is associated with chronic inflammation and aging-related diseases, such as cardiovascular diseases and type 2 diabetes [36,37]. Conversely, cancer cells have the ability to undergo unlimited cell division as they possess a mechanism to maintain telomere length [38]. In most cancer cells, telomerase activity, which is mediated by a ribonucleoprotein consisting of the telomerase RNA (TER) against telomeric DNA sequences and telomerase reverse transcriptase (TERT), is found to be increased, thereby supporting telomere elongation [39]. However, in other cancer cells, telomere elongation relies on a homologous recombination-based alternative lengthening of telomeres (ALT) pathway, which operates in a telomerase-independent manner [40].

**Figure 3:**
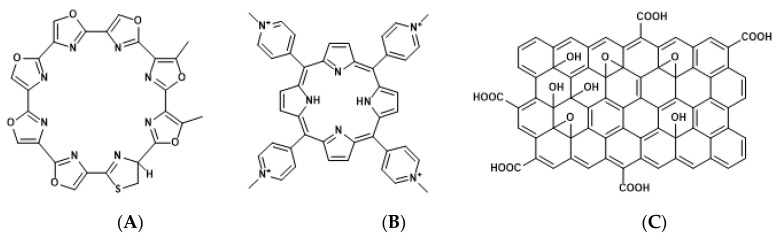
Chemical structures of telomestatin (**A**), tetra-meso (N-methyl-4-pyridyl) (TMPyP4) (**B**), and graphene oxide (GO) complex (TMPyP@GO) (**C**).

Human telomeric DNA consists of tandem hexanucleotide TTAGGG repeats with double- and single-stranded 3′-overhangs, which form a telomere loop (t-loop) by penetrating the 3′-overhang of the upstream duplex region [41]. The displacement loop (D-loop) corresponds to the area where the penetration of 3′-overhang occurs, and is required for the stacking of G4 [42,43]. G4 formation prevents the access of telomerase to telomeres. Therefore, telomerase activity was found to be suppressed by the G4-stabilizing ligands, such as telomestatin; TMPyP4 [tetra-meso(N-methyl-4-pyridyl)]; and its graphene oxide (GO) complex, TMPyP@GO (Figure 3) [44,45]. Conversely, ALT is facilitated by G4 stabilization [46]. Altogether, G4-stabilizing ligands targeting telomeres exhibit cytotoxicity toward cancer cells in which the telomeres are elongated through telomerase but not ALT. 

### 3.2. RAS

RAS family comprises three proto-oncogenes, including *KRAS*, *HRAS*, and *NRAS*, which encodes small guanosine triphosphatase (GTPases) that are expressed ubiquitously [47,48,49]. The sequences and structural features of these three RAS proteins are highly conserved [49]. In response to extracellular signal stimuli, such as growth factors and hormones, RAS protein becomes activated guanosine triphosphate (GTP) bound form via the receptor tyrosine kinase (RTK) and G protein-coupled receptors [47,48,49,50]. RAS protein then acts as a hub protein to control various downstream signaling pathways, including the phosphatidylinositol 3-kinase (PI3K) and mitogen-activated protein kinase signaling pathways [51]. In many cancer cells, the hyperactivation of RAS protein is known to be constitutively induced [52]. Missense mutation sites in the three hotspots of *RAS* are at codons G12 and G13 of exon 2 and Q61 of exon 3 [49]. These mutations lead to conformational changes resulting in the constitutive activation of RAS protein by the enhancement of binding to GTP and impairment of binding to GTPase-activating protein (GAP), which further catalyzes the hydrolysis of GTP to guanosine diphosphate (GDP) [49,53]. In addition to the missense mutations, amplification of the *RAS* gene causes hyperactivation [54]. Furthermore, hyperactivation of RTKs through coding gene mutations and increasing the amount of growth factors leads to aberrant RAS activation [55]. As the hyperactivation of RAS protein is closely associated with cancer cell characteristics, such as abnormal proliferation, survival, and invasion, it has been widely believed that small molecules targeting RAS have high potential as anti-cancer drugs [56]. However, it is extremely difficult to identify the inhibitors of RAS protein owing to a lack of proper binding pockets for small molecules and the high affinity of RAS protein toward GTP and GDP [57]. RAS protein has been considered as an undruggable target in cancer.

**Figure 4:**
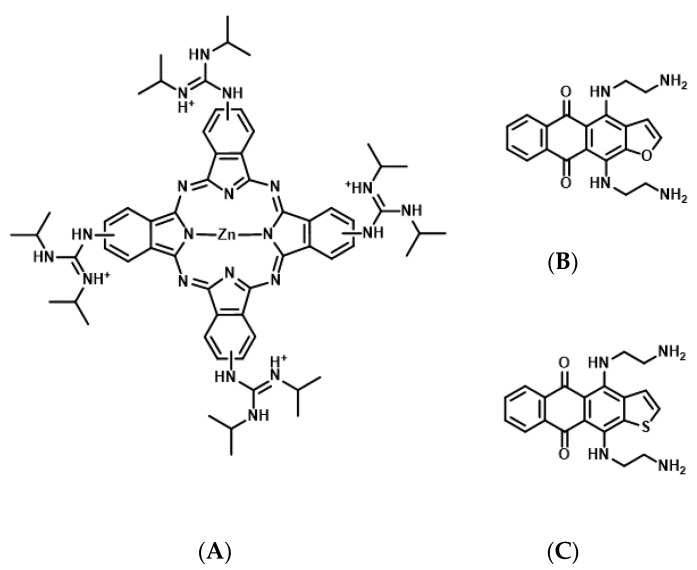
Chemical structures of tetrakis-(diisopropyl-guanidine) phthalocyanine (Zn-DIGP) (**A**), anthrafurandiones (ATFD) (**B**), and anthrathiophenediones (ATPD) (**C**).

G4-forming sequences exist in the promoter and 5′-UTR of all three RAS proteins and mRNAs, respectively (Table 1) [58,59]. Results of an in vitro transcription assay revealed that the formation of DNA G4 in the promoter hampers the transcription reaction of RNA polymerase [22]. Consistently, treatment with G4 stabilizing ligands, a cationic phthalocyanine tetrakis-(diisopropyl-guanidine) phthalocyanine (Zn-DIGP), and TMPyP4, targeting the promoters of *KRAS* and *HRAS,* decreased *RAS* expression in cancer cells (Figure 4A) [59,60]. Conversely, it has been demonstrated using in vitro translation and luciferase assays that the formation of RNA G4 in the 5′-UTR of *NRAS* mRNA decreases its own translation [61], further implying that RAS expression can be downregulated through the formation of RNA G4s, which prevents ribosomal elongation. This is further supported by the evidence that anthrafurandiones (ATFD) analogues [4,11-bis(2-aminoethylamino)anthra [2,3-b]furan-5,10-dione] and their analogs, anthrathiophenediones (ATPD) [4,11-bis(2-aminoethylamino)anthrax [2,3-b]thiophene-5,10-dione], repress translation by targeting *KRAS* mRNA, which strongly decreases the expression of KRAS protein in cancer cells (Figure 4B,C) [61].

## 4. Potent G4 Ligands as Photosensitizers for Molecularly Targeted PDT

### 4.1. Porphyrin Derivatives as Photosensitizers for Molecularly Targeted PDT

**Figure 5:**
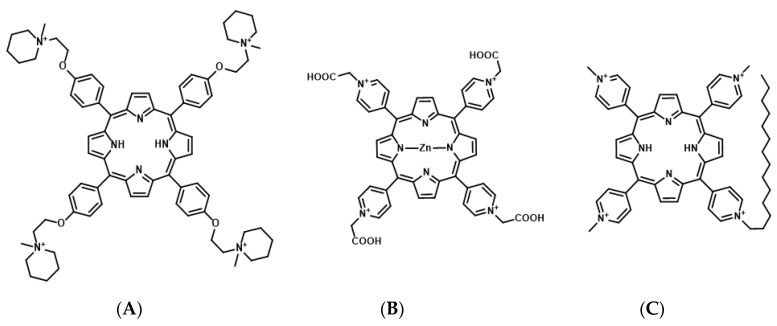
Chemical structures of 5,10,15,20-tetra-{4-[2-(1-methyl-1-piperidinyl)ethoxy]phenyl} porphyrin (TMPipEOPP) (**A**), Zn(II)-5,10,15,20-tetrakis(N-carboxymethyl-4-pyridinium)porphyrin (ZnPI) (**B**), and tri-meso(N-methyl-4-pyridyl)-meso(N-tetradecyl-4-pyridyl (TMPyP4-C14) (**C**).

Porphyrins are composed of a conjugated macrocycle of four pyrroles. Porphyrin derivatives are currently the most commonly used photosensitizers for PDT [62,63]. Many studies have assessed porphyrin derivative-based G4 ligands as photosensitizers for mtPDT. The porphyrin derivatives 5,10,15,20-tetra-{4-[2-(1-methyl-1-piperidinyl)ethoxy]phenyl} porphyrin (TMPipEOPP) and Zn(II)-5,10,15,20-tetrakis(N-carboxymethyl-4-pyridinium)porphyrin (ZnP1) have been identified as photosensitizers targeting telomeric DNA G4 (Figure 5) [64,65].

TMPipEOPP is a cationic porphyrin derivative that has four large side arms, which prevent intercalation to duplex DNA [64]. An in vitro study revealed that TMPipEOPP selectively binds to telomeric DNA G4 and cleaves it upon photo-irradiation through ROS production, resulting in cancer cell death [66].

ZnP1 is a cationic porphyrin derivative that binds to the DNA groove [67]. It selectively binds to the telomeric DNA G4, and the photo-activated ZnP1 generates singlet oxygen when exposed to irradiation. It further induces the oxidation of guanine residues at the telomeric DNA G4 and cleavage [65].

Although the effect of G4-stabilizing ligands targeting telomeric DNA on cytotoxicity depends on the telomere elongation system, telomerase or ALT, TMPipEOPP, and ZnP1 still exhibit cytotoxicity toward cancer cells.

The porphyrin derivative, tri-meso(N-methyl-4-pyridyl)-meso(N-tetradecyl-4-pyridyl (TMPyP4-C14), is a photosensitizer targeting RNA G4s of *KRAS* and *NRAS* mRNAs [59,68]. In TMPyP4-C14, the four methyl groups of the cationic porphyrin derivative are replaced with tetradecyl alkyl chain (C_14_H_29_) to improve the intracellular uptake efficiency by the cell membrane permeability [68]. In vitro studies have demonstrated that TMPyP4-C14 selectively binds to RNA G4s from *KRAS* and *NRAS* mRNAs, and photo-activated TMPyP4-C14 induces the cleavage of these RNA G4s through ROS production. Concomitantly, in cancer cells, TMPyP4-C14 decreases the expression of both *KRAS* and *NRAS* mRNAs and induces cell death upon photo-irradiation.

### 4.2. Phthalocyanine Derivatives as Photosensitizers for Molecularly Targeted PDT

Porphyrins exhibit strong absorption at a wavelength of approximately 400 nm (Soret band) by the π-electron system and weak absorption at wavelengths > 500 nm (Q-bands); however, phthalocyanines exhibit strong absorption of the Q-band between 600 and 750 nm, which is attributed to a benzene ring fused to the β-positions of pyrroles [58,62,63]. Therefore, phthalocyanine derivatives are thought to be prominent photosensitizers.

**Figure 6:**
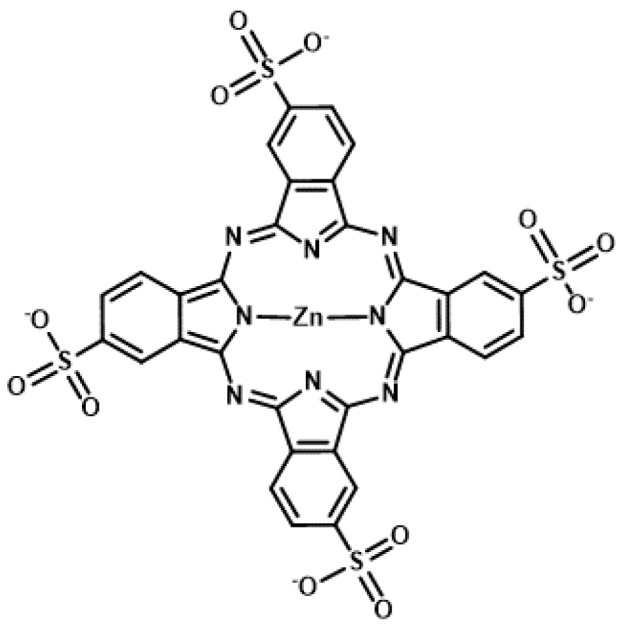
Chemical structure of zinc(II) phthalocyanine 3,4″,4″,4′″-tetrasulfonic acid, tetrasodium salt (ZnAPC).

The phthalocyanine derivative, zinc(II) phthalocyanine 3,4″,4″,4′″-tetrasulfonic acid, tetrasodium salt (ZnAPC) (Figure 6), is an anionic phthalocyanine generated through sulfonation, which acts as a photosensitizer targeting RNA G4 of *NRAS* mRNA [69,70]. In vitro studies have shown that ZnAPC induces ROS generation and RNA G4 cleavage from *NRAS* mRNA upon photo-irradiation. However, it is noteworthy that ROS is not required for cleavage in this context. The results of fluorescence lifetime measurements revealed the transfer of electron from the photo-activated ZnAPC to RNA G4, indicating that ZnAPC directly cleaves RNA G4 upon photo-irradiation. This reaction is a type I reaction of PDT, whereas most photosensitizers undergo type II reactions to induce cell damage. Conversely, it was proposed that radical species generated through the type I reaction lead to an amplified PDT effect, especially under low-oxygen conditions [71]. Consistently, ZnAPC has been shown to downregulate *NRAS* mRNA and NRAS protein expression and induce cancer cell death upon photo-irradiation, even in the presence of an ROS scavenger, N-acetyl cysteine [69]. Direct cleavage, which is independent of oxygen species, will cause ZnAPC to induce toxicity in cancer cells under low levels of oxygen (the state of hypoxia), which often causes therapeutic resistance and metastasis. Therefore, ZnAPC is considered a promising photosensitizer to expand the scope of application of PDT for deep tissues and large solid carcinomas. Moreover, disulfonated zinc phthalocyanine (zinc(II) phthalocyanine disulfonic acid, disodium salt) induces cell death upon photo-irradiation even under low levels of oxygen [72]. This supports the possibility that ZnAPC can effectively eradicate cancer cells under hypoxic conditions.

## 5. Conclusions

As shown here, there are a very limited number of G4 ligands that can be utilized for mtPDT, although there are a number of G4 ligands. As photosensitizers in mtPDT, G4 ligands should accumulate selectively in cancer cells to reduce their side effects on normal cells and to further enhance the medical efficacy of the targeted cancer cells. Moreover, as a molecularly targeted drug, a photosensitizer targeting G4 must selectively bind to G4 over duplex as most RNAs fold to form a duplex, which are abundant when compared to G4 RNAs. Additionally, many G4 ligands have been shown to induce DNA damage by stabilizing target G4s or by impairing helicases, although the molecular mechanism remains to be elucidated [73,74,75]. These G4 ligands cannot be used as photosensitizers as they exhibit cytotoxicity in the absence of photo-irradiation, even though they generate ROS upon photo-irradiation. In this review, we introduced G4 ligands, which can be utilized as potential photosensitizers for mtPDT. TMPipEOPP and ZnP1 have been shown to target telomeric DNA, whereas TMPyP4-C14 and ZnAPC target the *RAS* mRNA. These G4 ligands show high selectivity toward specific target(s) and cleave them upon photo-irradiation, whereas the cleavage mechanism is dependent on the photosensitizers. Chemical environments, including the concentration of oxygen and cations surrounding the DNA and RNA in cancer cells, are altered during tumor progression. Therefore, it might be necessary to use these photosensitizers for mtPDT considering their high affinity and selectivity of binding and cleavage, type of cancer, and structure and stability of the target G4(s) in cancer cells. Moreover, the use of G4 ligands, which can cleave target G4s directly in oxygen-independent type I reactions in mtPDT, could be promising for targeting deep-tissue cancers and large solid carcinomas.

Recently, research on the development of a robust system for the delivery of photosensitizers towards cancer tissue has been actively conducted [11,76,77]. Particularly, the surface-specific modification of photosensitizers using biomolecules, such as proteins, peptides, nucleic acids, or supramolecular architectures of photosensitizers, including dendrimers, micelles, and liposomes, is being well studied [78,79]. However, further studies to improve the delivery of G4 ligands in mtPDT are required for clinical application. Although the affinity of a ligand with a target G4 has been used as the standard index for the development of G4 ligands, which includes structure–activity relationship (SAR) studies and virtual screening of G4 ligands [80], it is now required to perform these studies using another index, such as structure selectivity of G4 ligands. Furthermore, combining different delivery systems and new G4 ligands may advance the practical application of G4 in mtPDT.

## Figures and Tables

**Table 1:** G4 forming sequences derived from *RAS* promoter and mRNA.

**Promoter**					
**Gene**	**Strand**	**Position from TSS**		**Ref of TSS**	**5′- Sequence -3′**
*HRAS* (NG_007666.1)	coding	207	231	NM_005354 NM_176795 NM_001130442	GGGCCGGGGGCGCGGGGCCGGCGGG
		264	281		GGGTGGGGCCGGGCGGGG
	non-coding	−412	−437		GGGCTACGGGCTGGGGAAAGGCTGGG
		−145	−171		GGGCGGGGCTTCCGGGAGCAACGCGGG
		19	-4		GGGTTGCGGGCGCAGGGCACGGG
		88	68		GGGGCGGGGCGGGGGCGGGGG
*KRAS* (NG_007524.2)	non-coding	−227	−261	NM_004985 NM_033360	GGGGTGGCTGGGGCGGTCTAGGGTGGCGAGCCGGG
		−176	−208		GGGCCGGGCCGGGCCGGCGGGGGAGGAGCGGGG
		−118	−145		GGGCGGTGTGGGAAGAGGGAAGAGGGGG
*NRAS* (NG_007572.1)	coding	−203	−176	NM_002524	GGGTCAGCTCAGGGGATGTGGGGGAGGG
		15	32		GGGAGGGGCGGGTCTGGG
**mRNA**					
**Gene**			**Position from AUG**		**5′- Sequence -3′**
*HRAS* NM_005343.4			−100	−81	GGCCUCGGCCCCGGCCCUGG
			18	28	GGUGGUGGUGG
			446	460	GGCAGGGAGUGGAGG
			586	602	GGACAUGGAGGUGCCGG
			634	647	GGAAGGAAGGACGG
			654	667	GGAAGGAAGGAAGG
*KRAS* NM_033360.4 NM_004985.5			−175	−165	GGCGGCGGAGG
			−157	−138	GGCGGCGGCAGUGGCGGCGG
			−145	−126	GGCGGCGGCGAAGGUGGCGG
			−133	−123	GGUGGCGGCGG
			-59	−49	GGCGGCGGCGG
			28	44	GGAGCUGGTGGCGUAGG
*NRAS* NM_002524.4			−240	−223	GGGAGGGGCGGGUCUGGG
			18	29	GGUGGUGGUUGG
			2261	2276	GGAUUUGGAGGCUUGG
			2953	2963	GGAGGUGGAGG
			3935	3948	GGAGGGAGGGGAGG
